# Transactions of the Medico-Chirurgical Society of Edinburgh

**Published:** 1824-06-01

**Authors:** 


*Transactions of the Medico-Chirurgical Society of Edinburgh.* Vol. I. 8vo. pp. 697, four coloured Plates. Edinburgh, 1824.

IT has long been a matter of surprise, that the INTELLECTUAL CITY should send forth no medical transactions, and but one medical journal—while the City of *Shop-keepers* should send forth four series of cotemporary medical transactions, and, at least a dozen of medical journals! Perhaps, on reflection, this will not be found attributable to want of will or power in the modern Athenians, but to the limited number of their population and the small scale of their public institutions, which do not afford materials for annual volumes of transactions. Books of this nature must be charged with practical facts rather than ingenious speculations and reasonings. If the latter would pass current in the world at large, Edinburgh might cope with London. But the enormous extent of the metropolitan population, hospitals, and infirmaries, must ever give the profession of this city a decided advantage in collecting facts, making observations, and trying experiments, which are now the only species of medical information that will at all be listened to or tolerated.

Still, with all these "ways and means" of the metropolis, the prodigious increase of medical journals within these few years, must excite surprise, mixed with some curiosity, to learn the causes of this multiplication. Although, in literary as well as commercial speculations, the supply will often surpass the demand; yet, in both instances, it must always be an increase of demand that creates the increase of supply. Throughout all ranks of the public at large, as well as of the profession, there exists a voracious appetite for novelty, which cannot be appeased by precarious or uncertain meals—but by a regular *periodical* supply;—the intellectual banquet being looked for with as much impatience as the savoury provender of the table. Under such encouragement, and considering the number of spare hands now in the world, is it wonderful that there should be an impetuous rush of intellectual wares into this new channel of consumption, in the same way as there was a rush of merchandize, a few years ago, into South America? True it is that, in both instances, the market has been glutted—but speculation is equally alive—and every failure that takes place will only give origin to a new enterprize.

Medical societies have multiplied with books and journals, though not, perhaps, in the same proportion. The advantages resulting from associations of medical men, where observations are communicated, opinions interchanged, and facts recorded, have long been duly appreciated by the profession at large. It is with the knowledge of medicine as with knowledge of the world—they are best acquired, or at least, perfected, by freely mixing in the society of our neighbours, where the angles and asperities of individual opinion and prejudice are gradually rubbed off by collision with those of others. In medical societies, besides the acquisition of knowledge, young men acquire a habit of arranging their ideas, and a facility of delivering them, which are of great service to them in their passage through life. Edinburgh was not deficient in debating societies—but the present is on a very different principle—that of recording and publishing the communications of those actually practising the profession, in imitation of the Transactions of medical societies in London and Dublin. This "first offering" from Edinburgh, is distinguished more by the respectability of the contributors, than the intrinsic value of the communications—a character which, we apprehend, will attach to every subsequent volume from the same source, and for the reasons which we before stated—namely, the celebrity and number of cultivators as compared with the narrowness and sterility of the soil. There is a hint thrown out, however, in the preface, which may have some effect, and our notice of it may contribute a little to second the intention of the Society. "From the number of medical men, in all parts of the world, who have been initiated into their profession in this University, it may be hoped that the Society may become the depository of much more varied and extensive observations than the resident members themselves can hope to furnish." We hope and trust that this invitation will not be unproductive, more especially in the early annals of the Society, before self-interest and cabal begin to influence its councils and destroy all equitable arrangement of the communications submitted to the decision of its committees. Neither do we think that this appeal to the quondam students of Edinburgh will be made in vain. No one, we think, can have spent a portion of his youth in that romantic city, without having received impressions on the plastic mind, that must ever afterwards respond to the reminiscences of former scenes and associations, whether fortune expose him to the fervor of an equatorial sun, or the chilling influence of a Polar sky.

> Pone sub curru nimium propinqui
> Solis, in terra domibus negata:
> Dulce ridentem Lalagen amabo
> dulce loquentem!

The volume before us contains twenty-six communications, of very unequal extent and importance. Among the contributors, we find the names of Duncan, Abercrombie, Kellie, Ballingall, Combe, Allison, Russell, &c.—names well known to the professional ear—and not more known than respected.

### ART. 1.

### *Contributions to the Pathology of the Heart.* By JOHN ABERCROMBIE, M.D.

Few men, excepting Morgagni, Bonetus, or Lieutaud, have published so many *cases* of disease as Dr. Abercrombie. The motto to his writings might, with very little straining of the sense, be the declaration of Æneas—

> Per *varios casus*, per tot discrimina rerum
> Tendimus.

We have heard objections made to this multiplication of cases and paucity of general principles—but we do not join in the objections. We believe that, for a long time to come, the plan of Dr. Abercrombie must be pursued. Indeed, we greatly doubt whether many general principles will ever be *established* in medicine—for as the organized beings, animal and vegetable, which have existed on this globe at various epochs, appear to have sprung up of different shapes, sizes, and structures, according to the state of the circumambient elements, so the diseases of man have varied, and will ever vary, from new causes and changes springing up, or going forward in himself, and in the moral and physical circumstances around him.*

Dr. Abercrombie divides the pathology of the heart into four classes—Inflammatory Affections—Organic Affections—Rupture—Displacement. Between the first two classes, there is, in fact, no real distinction—for the *first* includes the principal organic diseases—and the *second* is merely meant "to introduce a few cases, which appear to present some phenomena *differing a little* from the ordinary cases of organic disease of the heart."

---

* We see the breadth of a single mountain form the boundary between health and disease—nay, between two different states of organization. Thus, in the Valley of Chamouni, we find very few cretins or goitres; while, if we cross the Col de Balme into the Valley of the Rhone, we see hardly any thing else than idiotcy and bronchocele. The inhabitants of Grindelwalden and Meyrengen, separated only by the Grand Scheidec, present quite a different complexion and physiognomy. Examples of this kind might be multiplied ad infinitum.

I. *Inflammatory Affections.*—The distinction between inflammation of the pericardium where it is unattached, and where it is reflected over the heart, is, of course, nearly, if not quite impossible to be made in the living subject. Both forms often approach insidiously at first, and even complete accretion of the two sheets of membrane will take place before the disease is suspected. Both acute and chronic pericarditis are formidable diseases. We must considerably abridge Dr. Abercrombie's illustrative cases, and pass many of them over entirely. The following is introduced as a specimen of acute pericarditis.

*Case* 1. A young lady, 16 years of age, 8th January, 1812, presented the phenomena following, viz. acute pain at the pit of the stomach—greatly impeded respiration— extreme anxiety and restlessness—pulse 120—130—no cough or vomiting. Bled freely for several days in succession, with blisters, digitalis, &c. but with little effect, as the disease continued unabated for nearly a fortnight, until active treatment could no longer be pursued. In the third week the pain abated, and the breathing became freer. She then fell into a state resembling chorea, with delirium, during which she did not complain of any pain, and the breathing was natural. These symptoms subsided in a few days, and she gradually recovered her usual health, excepting some frequency of pulse. She continued well till the 20th April, when, after exposure to cold and fatigue, the original symptoms returned, *the pain being more in the region of the heart.* Depletion relieved, but did not conquer the disease, and she died on the 26th of the same month.

*Dissection.* Adhesion of the pericardium to the heart, in toto, by means of a thick layer of coagulable lymph, which was *soft and easily separable.* On the *outer* surface of the pericardium there was a similar deposition, nearly half an inch in thickness in some places. The surface of the heart very vascular—lungs inflamed and, in some places, indurated—the other viscera healthy.

Dr. A. has not stated whether the external exudation was of a firmer consistence than the internal. We have little doubt, that it was from the former inflammation the patient first suffered, and apparently recovered. The internal inflammation of the pericardium was of a much more formidable nature, and proved fatal.

*Case* 2. A boy, aged seven, had acute rheumatism in February 1819, with symptoms of carditis, both of which were relieved by copious bleeding. He continued well till November, when pain in the left side, cough, dyspnœa, and fever, were met by active depletion, which relieved the local symptoms, but the pulse continued very quick. There was no unusual pulsation in the region of the heart—tongue clean, appetite good. In this state he continued for a week. About the 20th day from the attack, he complained of pain in the left side, increased by pressure on the upper part of the abdomen. Local and general bleeding relieved this pain. Next morning he had a convulsion, and died in the evening.

*Dissection.* The abdominal viscera and the lungs were sound. There was adhesion of the pericardium to the heart throughout—the adhesion consisting, in some places, of a soft gelatinous matter—in others, of a firm reddish substance, like that of healthy granulations—the whole being soft, and easily torn by the finger. When cleared away, the surface of the heart was covered with firm, irregular elevations, like small granulations.

The *third* case is nearly similar to the above, and need not be detailed. We may here remark, that fatal affections of the heart, whether in young or old, are very rarely so masked as in the foregoing case. An attentive examination of the functions of this organ, and its action within the chest, will very generally reveal the lesion in question.

*Case* 4. This case is introduced by Dr. Abercrombie to shew that carditis may be going on insidiously in the course of another disease, by which it is, as it were, masked for a time.

A girl, 12 years of age, had erysipelas on the face, not severe, and confined to one cheek, but with considerable fever, and obstinate constipation. For several days the bowels resisted medicine, but about the 12th day the disease appeared to give way. About the middle of this period, however, Dr. Abercrombie found the little patient complaining of pain in the left side, which disappeared soon, and the breathing was unaffected. By the 14th day, the erysipelas had terminated by desquamation of the cuticle, and the fever had subsided—the tongue clean—the appetite returning. On the 15th, things took a turn for the worse, Her pulse became slow and irregular—the body cold—she died on the same day.

*Dissection.* The brain, lungs, and abdominal viscera healthy—the pericardium distended with about twelve ounces of a turbid, milky fluid, with flocculent matter floating in it. The whole inner surface of the pericardium, and the outer surface of the heart covered by a uniform coating of coagulable lymph of considerable thickness.

That this disease was, at least, coeval with the erysipelas, there can be no doubt—indeed, we are confident, that it long preceded the last-mentioned affection. We, therefore, suspect a laxity of observation in the case, otherwise the carditis would have been detected, or, at least, suspected, before the last day of the patient's life. Many of Dr. Abercrombie's cases, indeed, are very imperfect sketches, as far as regards the history of the disease—being mere "on dits" of the patient, or previously attending practitioner, Dr. A. only coming in at the close of the malady. We shall take the first case that offers as an example.

*Case* 5. " A man, aged twenty-three, died after an illness of seven months, in which the symptoms were *rather obscure*. When I saw him a short time before his death, he was much emaciated, but without any cough or expectoration. He had some pain in the left side, and at times a degree of dyspnœa, with quick breathing, but it was neither constant nor severe; and, when asleep, his breathing was quite natural. His appetite was good, and bowels natural; his pulse generally 100, or above it, but quite regular. The most prominent symptom was a strong pulsation of the heart, which was constant and violent. He died gradually exhausted, without any other symptom.

" *Dissection.*—The pericardium adhered intimately to the heart through its whole extent. It adhered also to the neighbouring lung. The right lung was adhering, and slightly hepatised, and much gorged with blood. On cutting into it, a cavity was laid open, the size of a pigeon's egg, full of watery fluid. The left lung was much loaded with frothy mucus. The spleen was enlarged, and tubercular." 14.

Here we have only the words "*rather obscure*" for almost the whole history of the case. Who can doubt, but that an accurate investigation of this young man's malady, by percussion, auscultation, and a thorough examination of the functions, would have revealed disease of the heart and lungs long before the period embraced by Dr. Abercrombie's account?

Our author justly observes that, when inflammatory affections of the heart are not speedily fatal, as in the above cases for example, they frequently terminate by adhesion of the pericardium to the heart, the disease then passing into the chronic state, and being protracted for a longer or shorter period according to circumstances. Although in some cases of this kind the symptoms are obscure and undefined, yet, in general, we find the action of the heart violent, with more or less embarrassment in breathing, and, at last, dropsical effusions, gangrene of the extremities, or death from gradual exhaustion.

" This highly dangerous and insidious affection occurs to us most frequently in connexion with rheumatism; but it may also supervene upon any other febrile disease, or it may come on in an idiopathic form, without any previous disorder. In its connexion with rheumatism, it may either attack when the rheumatic inflammation has suddenly receded, or it may appear at the termination of the disease, when the rheumatism has yielded in the ordinary way; or it may appear without any change in the rheumatic symptoms, and both complaints go on together. The symptoms vary considerably in different cases. In some there is pain in the region of the heart; in others none. The breathing is generally more or less oppressed, but sometimes in a slight degree; while, in other cases, the oppression is so sudden and violent, as to prevent articulation, and threaten instant death. There is, in general, strong pulsation of the heart; in many cases so violent, that it can be felt over every part of the thorax, and even by the hand laid upon the abdomen. In some cases there is vomiting, and in some cough; but cough is not a regular symptom. The pulse is generally frequent, sometimes extremely so; but in other cases it is little affected, and it has even been observed below the natural standard. Respiration is sometimes hurried, but not uniformly so; for in a severe case mentioned by Dr. Wells, in which the pulsations of the heart were 190 in the minute, respiration was soft and easy, and not above 24. Attacks of syncope sometimes occur; and in one case which has been communicated to me, syncope was induced by pressure upon the spot corresponding with the apex of the heart." 16.

A very small proportion of cases have, as yet, terminated favourably—yet it is not so uniformly fatal as it was twenty years ago. The prognosis, however, may generally be unfavourable, and, at the best, very guarded; for, even under the most judicious practice, when the disease appears to be subdued, the adhesion of the pericardium to the heart may have taken place, and the evil prove irremediable.

"The disease occurs in a more chronic form, in which it steals on slowly and insidiously without exciting much alarm. This form also may supervene upon rheumatism, or may come on without any previous disorder. After a rheumatic attack, or after slight inflammatory symptoms like a severe cold, the patient begins to feel a pulsation in the region of the heart, more or less violent, at first probably not constant, but excited by any exertion; the attacks being accompanied by some degree of breathlessness, a feeling of suffocation, and sometimes a remarkable throbbing in the head. The affection goes on gradually increasing,—the pulsation becomes permanent,—the attacks of dyspnœa more frequent and severe; till at length dropsical symptoms appear. In some cases the pulsation is not perceived, while the patient is lying perfectly still in the horizontal posture, but is excited by the least exertion, or by rising into the erect posture. On dissection, in both forms of the disease, we generally find the extensive adhesion of the pericardium to the heart, or sometimes, though more rarely, both surfaces covered with coagulate lymph, without adhesion. In the cases which have gone on for a considerable time, the heart is generally enlarged. This enlargement seems to be in many cases confined to the left ventricle, and to consist of an enlargement of the cavity, without thickening of the parieties. In a case by Dr. Baillie, it contained ℔ij. of blood. The substance of the heart is generally found pale, soft, and flaccid; and, in some cases, the effects of inflammation have been found in the cavities, as deposition of coagulable lymph within the ventricles, or about the valves, thickening of the valves, and tubercles, or projections of various sizes, on the inner side of the auricle or ventricle." 18.

*Case* 6. A young man, aged 11, experienced a rheumatic attack in June 1817, the affection being accompanied by strong palpitation of the heart. The rheumatism confined him several weeks and then subsided, the strong action of the heart still continuing. In the winter of 1817 he was confined, (but from what symptoms Dr. A. does not inform us) and the next summer (1818) was taken to Bath, where he was weak and felt much oppressed, but the only distinct symptom was palpitation of the heart. Again he was confined in the winter of 1818, and in the spring of 1819, seemed to recover perfect health, going to dancing schools, and taking much exercise—*but still the inordinate action of the heart continued.* During the summer of 1820 he continued apparently well; but, in the autumn, he began to have some difficulty of breathing, and some œdema of the ancles. These symptoms were relieved by medical treatment; but he continued in ill health all the winter, unable to use any exertion. In the summer of 1821 he again improved much, and in the winter of that year was able to attend the university—the pulsation still continuing in the thorax. In April 1822 he was seized with rheumatism, accompanied by increased pulsation of the heart, and soon followed by dyspnœa and anasarca; and now the disease made more rapid progress, till June and July, when he again rallied for a short time, but fell back again, and died in the beginning of September.

*Dissection.*—"On opening the thorax, the heart appeared of enormous size, seeming to fill more than half the cavity, and pressing the lungs upwards. The pericardium adhered intimately to every part of the surface of the heart; and externally it adhered extensively to the diaphragm and the left lung. The enlargement of the heart was found to be entirely in the left auricle and ventricle, which were prodigiously enlarged and distended with dark grumous blood. The auricle was a great sac, containing at least ℔i, and the ventricle was enlarged in a still greater degree. Along the posterior part of this immense ventricle, the right ventricle was found lying in a collapsed state, a little elongated, but not diseased. The parietes of the left ventricle appeared rather thinner than natural, and the substance of it was pale, soft, and flabby. The aorta and its valves were healthy. The valves betwixt the left auricle and ventricle were thickened, and contained some nodules of bone. One half of the valve was considerably puckered and contracted; the other was elongated, and of a thick, firm, fleshy consistence. The aperture was quite free. The lungs were dark coloured, and much loaded with fluid. There was some fluid in the cavity of the pleura, and a small quantity in the ventricles of the brain." 22.

*Case* 7. The next case related by Dr. Abercrombie was more rapid in its course, though nearly similar in its nature. Cardiac affection followed two attacks of rheumatism in a young man, and resisted depletion and other means of relief. He died suddenly (three months from the commencement of the disease) in getting out of bed. On *dissection*, the left chambers of the heart were greatly enlarged—the auriculo-ventricular valve of that side imperfect and corrugated, so that it was incapable of shutting the communication between the two chambers—liver and spleen enlarged.

Dr. Abercrombie relates two cases "of the disease treated successfully." One of these, as it is short, we shall give in the author's own words.

*Case* 8. "On the 21st January 1817, at seven o'clock in the evening, I saw a man, aged twenty-four, who was affected with acute rheumatism, chiefly seated in his wrists and ankles, especially the latter, which were swelled, extremely tender, and highly inflamed. He complained of an acute pain in the region of the heart, increased by inspiration; his breathing was so oppressed and anxious, that he was unable to lie down, but was supported by pillows in a half sitting posture. The pulsation of the heart was so violent, that it was felt distinctly over every part of the thorax, and on the upper part of the abdomen. The pulse was 106, and intermitted about twelve times in a minute. The rheumatism had existed about a week; the pulsation of the heart had been felt for two days; the pain and dyspnœa had commenced on the morning of the 21st. A few hours before I saw him, he had been largely bled by Dr. Beilby; and we immediately bled him again to between 30 and 40 ounces. After two hours more we found him considerably relieved. Pulse 112, and regular. The ankles still highly inflamed, and extremely tender. He was again bled to 18 or 20 ounces, when he fainted completely, and lay for a long time in a state of extreme collapse; the pulse scarcely to be felt. The inflammation now completely left his ankles; and he could allow them to be moved or pressed in any way without uneasiness; his breathing was quite easy, and the pulsation of the heart had subsided. On the following day he was free from uneasiness, and continued well, except from slight rheumatic pains, without fever, which subsided in a few days." 28.

Upon this case we would remark that, there is a great difference between the simple extension of rheumatic inflammation to the heart, for the first time, and that organic change which afterwards goes on as an effect of inflammation. It is a comparatively easy matter to check or remove the first, but a very difficult task to do the same with the second. But, after all, is this a case of cure? It is precisely in the above circumstances that we find affection of the heart *follow*, at longer or shorter intervals, the cessation of rheumatism in rheumatic metastasis. The patient's subsequent health is, therefore, a matter of query.

The case 13, page 29, we believe to have been one of functional disorder of the heart in a female of arthritic habit, rather than inflammation of the heart's structure. This disordered action of the heart was not removed when the case closes. The patient was a lady, aged 58, of a full habit, who was seized on the 6th September with severe lumbago, "*unaccompanied by fever or any other symptom.*" The complaint subsided in five or six days, and, in a few days after its cessation, she felt a pain darting from the back to the breast and left side, with a sense of "*fluttering at the heart.*" This and palpitation, with irregularity of the heart's action, continued for several days, in spite of copious depletion and blisters. At length, the symptoms considerably subsided, after full vomiting produced by previous blood-letting carried to syncope.* Nevertheless, after nearly a year's interval, "*her pulse has been always irregular, and she is liable to strong action of the heart, upon any exertion or exposure to cold.*" We do not, therefore, consider this as a case of carditis—nor the cure complete. We may just remark, that the general blood-letting in the above case, appears to have been carried to an extreme extent, considering that there was no pyrexia—no appearance of inflammation in the blood abstracted. We have seen several cases of this functional disorder, and they were best managed by perfect quietude, and very gentle evacuations, succeeded by antispasmodics and anodynes.

Here our author makes some pathological observations on *active* and *passive* aneurism of the heart. The *former*, he thinks, arises from organic obstruction to the transmission of blood, or from such imperfection of the valves as allows the column of blood to act backwards upon the cavity whence it issued—"the muscular parts in both cases being in a sound and vigorous state." The enlargement, on the other hand, which supervenes on inflammatory action, appears to Dr. Abercrombie to be owing "*to an impaired condition of the muscular power produced by the inflammation, the cavity becoming incapable of emptying itself, and being gradually dilated by that impulse which, in the healthy state, would have excited it to contraction.*" To this pathology we cannot altogether subscribe. We believe there are few attentive observers who have not examined many cases where the inflammation was unequivocal, and yet the *active* aneurism or hypertrophy appeared on dissection. In short, our experience has led us to a contrary conclusion from that of Dr. Abercrombie—namely, that *inflammation* is far more frequently found in conjunction with *active* aneurism, than with *passive* dilatation of the heart. In three fourths of those cases of diseased heart, which follow acute

* No febrile symptom is adverted to in this report.

rheumatism, or its translation to the heart, we find the parietes of that organ *thickened*—although inflammation had been ravaging on its muscular structure throughout the disease.

According to Dr. Abercrombie's doctrine, we should, in these cases, find passive dilatation, which is but seldom the case comparatively. We agree with our author, that the term angina pectoris has been made to include several affections considerably different in their nature. It seems, also, to be admitted, that the disease in question cannot always be traced to any cognizable organic affection of the heart. The ossification of the coronary arteries, to which it has been referred by Dr. Parry and others, is known to be very often wanting; and to be present without symptoms of angina pectoris. Dr. Abercrombie sees no principle upon which the phenomena of syncope anginosa can be explained, "except a derangement of the muscular action of the heart."

"The term Spasm has sometimes been applied to them: the phenomena appear rather to favour the conjecture of diminished action, one of the cavities being loaded with blood, which it is unable to expel, and thus interrupting the harmony of the whole. It is probable that such a condition of the parts may occur as an incidental and temporary affection, and that, after continuing to recur for some time, it may be removed, and the parts be restored to their healthy relations. But, it is also probable, that after it has once taken place it may be more and more liable to recur from slight causes, until it terminate in permanent diminution of the muscular power of some part of the heart, and this be followed by permanent enlargement." 36.

We are inclined to agree with our author in a great measure, on the above point—and also on another, the probability that a slight degree of rheumatic inflammation, not sufficient to excite attention at the time, may lay the foundation for disease of the heart in some cases. It must be confessed, however, that the whole subject is involved in great obscurity. Dr. A. here introduces three cases, shewing angina pectoris with ossification of the coronary arteries—the disease without any such appearance—and extensive ossification without symptoms of angina pectoris. The second case is the most interesting, as presenting the phenomena of angina pectoris from imperfection of the valvular communication between the left auricle and ventricle. As the case cannot be abridged, we shall give it in Dr. A's own words.

*Case* 9. "A gentleman, aged fifty, of a full habit, consulted me first in July 1821. At that time he complained of an obscure uneasiness across the epigastric region, occasional palpitation, and a pain in the region of the heart, which attacked him at uncertain periods, especially if walking quick or up-hill. On these occasions it generally obliged him to stop; and if he attempted to persist in walking, vomiting was induced. In the night-time, he was occasionally seized with difficult breathing, accompanied by a pain extending from between the shoulders to the sternum, so violent as to oblige him to get out of bed immediately. His pulse was regular, and his general appearance that of robust health. The complaint was of two years standing. At its commencement, it came on suddenly while walking; and the first attack was accompanied by a sensation 'as if a fluid ran from the right side of the chest to the left side, and then stopped suddenly, passing through a space of three or four inches.'

"Soon after I saw him, he went to the Continent, where he thought himself considerably better. He returned to London in 1822, where he was frequently seized in the night with attacks of violent dyspnœa. He returned to Edinburgh in the beginning of winter. In the night of 23d December, he was seized with one of these attacks of dyspnœa, so severe as to threaten instant death. He was described to me by a surgeon, who saw him in the attack, as affected with the greatest degree of dyspnœa; his face livid; his body cold, and covered with a clammy sweat; his pulse scarcely to be felt. He was unable to speak, was nearly insensible, and appeared moribund. In this state he continued about two hours, and then gradually recovered. He consulted me again on the day following this attack. He was then in his usual health, and described his symptoms precisely as he had done in 1821, with the addition of these severe attacks of dyspnœa, which, however, had only occurred a few times, and at long intervals. He had also been lately seized, while in bed, with pain in the arms, extending across the chest, and accompanied by some dyspnœa, cough, and expectoration. He lay with greatest ease on his back. If he turned to either side, especially the right, he was apt to cough. His pulse was a little frequent, but regular; and his general appearance was still that of robust health. There was nothing unusual to be felt in the action of the heart. His urine was rather scanty. He was now put upon a regulated diet, with attention to his bowels, some diuretics, and confinement to the house, the weather being very cold. Under this plan he improved remarkably, and in the subsequent visits which I paid to him, he made little or no complaint. His nights were much better; his breathing easy; his pulse quite natural. My last visit to him was on the 16th January 1823, when he seemed in excellent health and spirits, and made no complaint, except that he was becoming very impatient of confinement. In the following night he was seized with a fit of dyspnœa, and was dead in a few minutes, before a sugeon, who lived in the floor above, reached his apartment.

"*Dissection.*—The lungs were much loaded with blood, but in their structure quite healthy. The right cavities of the heart were natural; the left auricle and ventricle were considerably enlarged, and loaded with blood; their parietes appeared about the natural thickness, except a small part near the apex of the heart, which was remarkably thin. All the valves were quite healthy; and no other morbid appearance could be discovered, except a remarkable enlargement of the opening betwixt the left auricle and ventricle. They had quite the appearance of one continued cavity, with scarcely any division; and it appeared evident, that the valve could have had but a very imperfect action in shutting the opening. There was some bloody fluid in the cavity of the thorax, and a slight appearance of ossification at the commencement of the aorta." 41.

The next subject which engages Dr. Abercrombie's attention, is what has been termed polypous concretions in the cavities of the heart. It is not now considered generally as a cause of disease, but as a deposition of fibrin in the act of dying, or very soon afterwards. We are by no means of opinion that this is always the case, for we have seen several instances where the concretion was so organized as to prove its having existed in the cavities of the heart long before death. Dr. Abercrombie is inclined, also, to attach more importance to these appearances than is generally done. "In the following case, (says he) it occurred in such a remarkable extent, that it could not be considered as an incidental occurrence."

*Case* 10. "A gentleman, aged sixty, had been liable for six years to palpitation of the heart and dyspnœa. After some time, he became dropsical; was often relieved by diuretics; but the dropsy always returned after various intervals, the intervals becoming shorter. Four years before his death, he was seized with hemiplegia of the right side, and his speech was considerably affected. From these symptoms he never recovered. When I saw him along with Mr. William Brown, a short time before his death, there was a strong and irregular pulsation of the heart; the pulse was weak, irregular, and rather frequent. He was liable to severe attacks of dyspnœa, and occasionally to fits of extreme faintness and coldness. He had general dropsy, palsy of the right side, and inarticulate speech. He died, gradually worn out by protracted suffering, in September 1818.

" *Dissection.*—There was extensive effusion, both in the thorax and abdomen. The pericardium adhered to the pleura costalis by a firm, narrow band, less than an inch in length. The heart was much enlarged. Upon opening the right ventricle, the cavity was found much enlarged, and completely filled and distended by a firm, solid mass of fibrin, of a light yellowish colour, without any appearance of blood. On removing the mass, which was of very great size, a small quantity of blood was found below it. The left ventricle was also enlarged, and was full of grumous blood. No disease could be discovered in the structure of the heart, after the most careful examination, except that the substance of it appeared paler than natural. The enlargement seemed to consist in the dilatation of the cavities, without increase of substance. The aorta was sound. The lungs were much loaded with fluid, and a little indurated. In the anterior part of the left hemisphere of the brain, a portion of the cerebral substance, the size of a large walnut, was of a brownish-yellow colour, and much indurated, except at its lower part, where it was approaching to suppuration." 48.

II. *Organic Diseases.*—It is evident that nine-tenths of the cases we have been considering were organic diseases; and, therefore, this division, "differing *a little* from the ordinary cases of organic diseases," appears to us quite useless. There is no class of diseases in which we do not find numerous cases differing, *not a little,* but a great deal, from the ordinary course; and the division of such would be endless. Under this head, Dr. Abercrombie has stated six cases. The following is the only one of these which we think it necessary to present to our readers.

"A young lady, aged eighteen. I was first consulted about her in September 1822, on account of anasarca of the legs. No other symptom was complained of; but, on examination, I discovered a remarkably strong and extended pulsation of the heart, of which I could obtain no history, except that she had been for several years liable to palpitation, but had experienced no inconvenience from it. The pulse was quite natural. The anasarca soon disappeared under the usual treatment, and she went to the country in her usual health. Some weeks after she had cough, with pain of the breast, and was bled from the arm with relief. She then made no complaint; but the strong and extended action of the heart continued. In the beginning of winter, she had again cough, and considerable expectoration, which had a puriform character. The pulse slightly accelerated, but quite regular. The strong action of the heart continued; and it appeared on close examination that it was not synchronous with the pulse, but seemed to alternate with it. She seemed to suffer little or no inconvenience from it. Through the winter she continued with little change, and no very urgent symptom. The cough varied; sometimes troublesome, and sometimes less so; the pulse regular, and very little above the natural standard. In the end of February she was out several times, but, in the beginning of March, the anasarca returned, with some hæmoptysis, dyspnœa, lividity of the countenance; and she died about the 20th, the action of the heart having continued as before, and the pulse nearly natural up to the day of her death.

"*Dissection.*—On opening the thorax, the pericardium appeared to extend over a much greater space than usual: it adhered closely to the left lung, and the right adhered closely to the mediastinum, so as to form one continued uniform surface. On the outer surface of the pericardium, there was some deposition of coagulable lymph. Internally it contained a good deal of fluid, and was free from any adhesion to the heart. The heart was much enlarged, and presented a singular appearance, being distinguished into two separate portions, one of them of a deep purple colour, the other of the usual colour. The former was the right auricle, so enormously enlarged as nearly to equal all the other parts of the heart; it was thin, and completely distended with dark grumous blood. The right ventricle was also much enlarged; the auriculo-ventricular aperture was large, and the valve corrugated. The left auricle was enlarged, and distended with grumous blood, and the auriculo-ventricular aperture was reduced to a narrow opening, by the thickening of the valve, and the adhesion of the parts of it to each other. The left ventricle was quite of the natural appearance, and the valves of the aorta quite healthy. The lungs were considerably hepatised; the left was much compressed by the enlarged heart." 57.

The principal lesions in this case were confined to the *right* side of the heart, which explains the "nearly natural state of the pulse."

III. *Rupture of the Heart.*—Our author has met with two cases of this dreadful accident, within the pericardium; and the symptoms were so different that one of them, Dr. A. avers, "might have been mistaken for apoplexy." The reader will, we think, be as much surprised as ourselves, when he peruses this case *resembling apoplexy.*

"A man, aged about thirty-five, had complained for some time of headach, but had not been confined from his usual labour, which was that of a joiner. One evening he had returned from his work, and was sitting by the fire, when, in stooping forward to lift something, he fell forward on the floor and expired." 60.

In the above case we do not perceive a single symptom of apoplexy—except the last—the *expiring*, which, we believe, is common to fatal cases of all diseases. As for *head-ache*, we have never observed it complained of *in* apoplexy, and it is well known to precede or accompany innumerable other diseases, so that it cannot be enumerated among the symptoms of apoplexy. In the foregoing case, all was found healthy in the head, but the heart was ruptured, and the pericardium distended with coagulated blood.

Dr. Abercrombie relates a case which occurred in the practice of Mr. George White, which is very remarkable, and of which we shall present the reader with the particulars. A man, aged 77, robust for his age, was at work as a labourer, on the morning of March 19, 1823, when he was suddenly seized with pain in the chest, extending from spine to sternum, accompanied by sense of great weakness. With difficulty he got home, when his pulse was found to be extremely small and feeble, but regular and not frequent. He lived ten days in this state, with very little change of symptoms. On dissection, the cavities of the pleura contained three pints of fluid. The lungs were sound.

The pericardium contained an immense quantity of coagulated blood. The aperture from which the hæmorrhage had taken place was in the left ventricle, and not larger, externally, than to admit the point of a catheter, but communicating, internally, with an ulcerated surface the size of a shilling.

IV. *Displacement of the Heart.*—Only two cases of this kind are related, and we do not deem them of importance.

We have now furnished our readers with a very full analysis of Dr. Abercrombie's paper, which will enable them to appreciate its practical value. Dr. Abercrombie's object was, of course, pathological; otherwise he had a wide field for observation on the various and extraordinary functional disturbances and physiological phenomena produced by organic affections of the heart. We part from this able physician and pathologist with the most perfect esteem and respect.

### Art. II.

*Remarks on the Cranium of a Man who died of Syphilis.* By George Ballingall, M.D. Professor of Military Surgery, &c. &c.

This short but interesting paper bears on a question of deep practical importance, by no means decided in the surgical world. We shall exhibit the prominent particulars of the communication.

In May 1822, Dr. B. visited a man, who was apparently near his end from syphilis or, as it is called, "the Poison of Mercury"—or pseudo-syphilis—or a mixture of all these, such is now the entangled state of the question! All that could be learned was, that the disease had commenced by an ulceration on the penis destroying part of the glans, followed by buboes, ulcerated throat, cutaneous eruptions, and exfoliations of bone from the nose. He had laboured under the disease for years—had been treated by several practitioners—had used much mercury and other remedies. He was now taking decoction of sarsaparilla and Plummer's pill, under the direction of Dr. Kenny. He was extremely emaciated—the face and upper part of the scalp covered with numerous blotches and incrustations, one of which projected like a horn over the centre of the frontal bone. Various superficial ulcerations and blotches were also conspicuous on his body and limbs. Under the medicines abovementioned he rapidly recovered, so as to be able to go about—and live irregularly. He caught cold—had a relapse, and died in a state horrible and loathsome to himself and others. The following were the appearances on dissection.

"On examining the exterior surface of the cranium, a circular portion of the right parietal bone, about the size of a shilling, may be observed flattened and somewhat rough; from this an exfoliation had taken place previous to the patient's coming under my care. On the interior surface of the bone, opposite to this spot, the impressions of numerous small vessels are to be seen deeply indented into the bone, and giving it a rough scabrous feel.

"On the central aspect of the frontal bone, two circular portions are to be seen marked by the impressions of numerous small vessels similar to what is observed on the parietal bone.

"Large portions of the superior maxillary bones, including the alveolar processes of the front teeth, are in the progress of exfoliation.

"On examining the base of the skull, the condyloid and cuneiform processes of the occipital bone, and the posterior clinoid processes of the sphenoidal bone, may be observed partially diseased." 71.

Of this case, Dr. B. observes, different views will be taken, according to the tenets of the practitioner. The patient will be considered by one class as poisoned by mercury—by another, as sacrificed for want of it. There is no doubt, however, that the disease, under which the patient fell, had a venereal origin, "and," says Dr. Ballingall, "from my never having observed a similar affection of the bones in any of those numerous cases in which I have exhibited mercury to a large extent, for the cure of hepatitis, I am naturally inclined to consider the morbid appearances which this skull presents as the result of the syphilitic poison." In support of this, he contrasts the above case with the three following.

"1. In the year 1812, while serving in the East Indies, Charles Forster, a soldier of the Royals, came under my care affected with venereal blotches and ulcerations on various parts of his body: he had considerable swelling of the nose and face, with an offensive discharge from the nostrils; and after the exfoliation of a portion of the vomer and inferior spongy bone, a discharge of about sixty or eighty maggots took place from the antrum. This patient was at the same time affected with phthisical symptoms, and, notwithstanding these, I was induced to persevere in the use of mercury, under which he recovered.

"2. In the course of last year, I saw a young lady several times, who was kept for *seven months* under the influence of mercury, for the cure of an obstinate liver disease, and eventually recovered, without any affection of the throat, skin, or bones.

"3. For the last *two years*, I have occasionally visited a lady labouring under extensive visceral disease, whose system had been for nearly the whole of the above period under the influence of mercury, and who, nevertheless, has not had the smallest symptom of mercurial disease,—no ulceration of the throat,—no eruption on the skin,—no thickening of the periosteum,—nor caries of the bones.

"It were easy for me to refer to numerous cases similar to the above, but at present this appears to be totally unnecessary; for while rotten bones have long been considered the legal inheritance of those who have suffered much from venereal disease, I am not aware that a *single instance* has yet been brought forward of mercury producing a similar affection of the bones, when exhibited for the cure of any other disease than syphilis; and until well marked and unequivocal cases of this kind shall be produced, those who advocate the utility and safety of mercury are not bound to obviate an objection to its use, which has not been proved to exist." 73.

Our own experience coincides with that of Dr. Ballingall.

### Art. III.

*Some Observations on a peculiar Affection to which the Bones of the Cranium are liable.* By James Russell, F.R.S. E. &c. &c. &c.

Our author observes, that the bones of the skull are liable to the diseases of other bones, as caries, erosion, exfoliation, &c. and, also, to an affection peculiar to themselves, namely, the complete separation of a portion of the flat bone from its neighbours, without any apparent disease, by a process of absorption. A similar process is, no doubt, employed by Nature, to separate the dead or diseased parts of the body from the sound—but here no disease apparently exists to give excitement to the absorbents for such operation.

"I first witnessed an instance of the separation of a portion of a sound parietal bone, in the case of a young man, who had a small tumour removed from the scalp by excision. The base of the tumour adhered to the pericranium, which had to be removed along with it; and by its removal, left a small portion of the cranium bare. But this very limited denudation of the cranium would not, under ordinary circumstances, have produced any perceptible effect; or at most, only the exfoliation of a superficial lamella of bone not thicker, nor more extensive, than a herring scale. But the separated portion to which I refer, was the whole thickness of the cranium, and equal in dimensions to a crown-piece. In all its circumstances, it possessed the character of a healthy bone, retaining the colour, thickness, weight, and every other appearance of health. No circumstance appeared in the history of this case, to account for the commencement of the separation of this portion of healthy bone, at so great a distance from the place where the irritation was applied. There was no useful purpose to be accomplished by the removal of this large portion of healthy bone; so that the cause from which this process originated is involved in complete obscurity.

"The next instance in which I had an opportunity to observe the commencement of a similar process, was in the case of a young lad, who had his skull fractured by a fall. He died in about twelve days after the accident, when the erosion was found to have begun at a considerable distance from the place where the injury had been received. The nature of the violence which a fall produces will not account for the establishment of the process of absorption, at so great a distance from the place of the original violence, since there is nothing in the nature of the violence itself, to affect the texture of the bone, and to excite irritation, at the remote place where the absorption began." 76.

From Mr. Russell's reasonings on these two cases, we have not been happy enough to glean any thing that elucidates the nature of them. They are, like many other anomalies in medicine and surgery—inexplicable.

### Art. IV.*

*On Dislocation of the Hip and Shoulder Joints.* By Adam Hunter, M.D. &c. &c. &c.

We owe the present paper of Dr. Hunter to the circumstance of his having dissected a *recent unreduced dislocation* of the hip-joint, and witnessed two of the shoulder; one recent, the other apparently of very old date—both of which had been reduced.

*Hip-joint.* There was an apparent difference of an inch in the length of the limbs, when the corpse was placed on the table, the dislocated limb being the shorter. The toes were turned inward—great enlargement and fulness of the upper part of the thigh and hip, completely concealing the trochanter. On separating the gluteus maximus from its various origins and its subjacent connexions, the head of the femur was brought into view, passing under the edge of the gluteus medius, lying deeply imbedded in coagulated blood, and bound down very firmly on the sacro-sciatic notch, by the inferior and posterior edge of this muscle, which passed over the neck of the bone. On clearing away the blood, Dr. H. found that the head of the femur lay between the pyriformis muscle and the great sciatic nerve, pressing the muscle against the superior and posterior part of the notch, and the nerve against the inferior and anterior. The nerve was quite flattened by the pressure.

"I now raised the gluteus medius, which set the thigh bone free, and likewise displayed such an appearance of destruction and confusion, as

* We have passed over Dr. Kellie's long and curious paper in the work under review, because we were unable to do justice to it at the close of an article. We shall not fail to take due notice of it in our next number.

it is impossible for language to describe. To say which was the attachment of the gluteus minimus, of the pyriformis, of the obturator internus, or in fact of any of the smaller muscles inserted into the vicinity of the trochanter, was impossible; but when I said 'I raised the gluteus medius which set the thigh bone free,' a suspicion of what had taken place must have occurred to every one's mind, viz. that all the small muscles near the joint, and the ligaments which belong to it, were torn from their attachments;—and this was literally the fact, as the head of the femur, which was previously kept fixed and immoveable by the stricture formed by the inferior edge of the gluteus medius, could now be made to move freely in every direction. Having removed the coagulated blood that lay on the surface of the gluteus minimus, I found that it was so much bruised and lacerated by the head of the femur passing over its surface, as to be reduced to a gelatinous or pultaceous mass." 173.

Dr. H. next directed his attention to the state of the ligaments, both of which he found completely torn from the head and neck of the femur. To bring the acetabulum into view, he cut across the large muscles that descend from the pelvis, at a short distance from the trochanter, thus separating the limb from the trunk. This afforded another proof of all the small muscles having been lacerated from their attachments. "The capsular ligament was, throughout its whole extent, attached to the circumference of the acetabulum, and the round ligament, quite entire, but slightly raised from its attachment, was lying in the bottom of the socket." In addition to these morbid appearances, there was a dislocation of the sacro-iliac synchondrosis; together with a fracture of the os innominatum of the right side, which traversed the acetabulum.

Dr. Hunter admits, what indeed is most obvious, that the preceding case is an extreme one, and cannot, therefore, be taken as a sample of the extent of injury to which the soft parts are liable in all cases of dislocation. Still, our author is inclined to think that, in many cases of this accident, the soft parts suffer to a much greater extent than surgeons are willing to admit. Here Dr. Hunter offers some strictures on the observations of Sir Astley Cooper on this point. But when we remember that the patient, in Dr. Hunter's case, was literally broken to pieces by the accident, whatever it was, it is quite impossible for us to take it as an example of the injury done to the muscles in dislocations. That this injury cannot be any thing like what occurred in Dr. Hunter's case, may be inferred from the speedy recoveries which generally take place when reduction is properly and early accomplished. We shall introduce the concluding passage from this paper.

"The above case, however, points out a new and additional force, exerted by one muscle, which has not been noticed by any previous writer that I have had an opportunity of consulting. The head of the femur, it will be remembered, had passed from beneath the inferior edge of the gluteus medius muscle,—the neck was firmly embraced by its margin,—the trochanter lay concealed behind its fleshy belly; and during life, while the stimulus of altered position existed, and the irritability of the muscle must have kept it in a state of continued violent contraction, the neck of the thigh-bone must have been embraced with immense force. Had reduction, therefore, been attempted, during the life of the individual, the head of the bone must have experienced a very powerful resistance to its return, from this cause. The stricture thus exerted by the margin of this muscle, passing over the neck of the thigh-bone, like a cord over a pulley, will, I think, most probably be the case in every instance of dislocation upon the sacro-sciatic notch, and consequently may be adduced, as a most admirable illustration of the propriety of that preparatory treatment, of lowering the tone of the muscular fibre, previous to the attempts at reduction being made, which has been so highly recommended, and practised with so much success, by Sir Astley Cooper." 178.

The dislocation of the shoulder, in the same individual, was similar to that of the hip, but with still more injury to the parts. There was a laceration in the belly of the deltoid muscle, fracture of the humerus, rupture of the tendon of the supra-spinatus muscle, of the capsular ligament; fracture of the coracoid process of the scapula, &c. &c. Surely Dr. Hunter would not adduce this as an example of the lesion of parts in shoulder dislocation. The same observation bears on this as on the former case.

### Art. V.

*On the Use of Tobacco in Tetanus.* By Thomas Anderson, M.D. Inspector of Health of Shipping, and Member of the Medical Board, Port of Spain, Trinidad.

We believe that more than one or two practitioners in this country have made trials of tobacco in tetanus, and apparently with success. Dr. Anderson resides in a climate where the disease is more prevalent than in this; his opportunities of observation are, therefore, numerous, and we hope he will prosecute a subject which, in this paper, is little more than broached. Tetanus is not so frequent in Trinidad as in some other of the West India Colonies, and less so in the town of Port of Spain than in the country. Negroes are more often the subjects of it than Europeans, and among them, males more commonly than females. Several cases of traumatic tetanus proving fatal under the ordinary modes of treatment, our author was led to try the effects of tobacco, which appears to be a remedy resorted to by the natives of the Spanish Main. The first case was that of a free negress, 40 years of age, who, having been cupped on the temples, with a blunt pen-knife and small calabash, became affected with trismus. Her pulse was accelerated, and there was some perspiration on the neck and chest. She was seen two days after the appearance of trismus. Dr. A. directed a strong decoction to be made of the fresh leaves of the indigenous Nicotiana Tabaccum, with which the jaws, throat, and chest were to be fomented for half an hour at a time. Then cataplasms of the same were to be applied to the jaw and throat. The warm bath, into which a quantity of the tobacco decoction had been thrown, was also administered every three hours, and a glyster of the same decoction to be thrown up every twelve hours. The bowels were ordered to be acted upon by calomel and gamboge pills. The usual effects of tobacco were produced, though not so decidedly as might be expected. Relaxation became general, the bowels were opened. The symptoms of trismus remained stationary for two days under this treatment. On the third day the jaw relaxed a little, and by perseverance in the plan, she recovered perfectly, though very slowly.

The second case was that of a negress also, who had one of her hands wounded by a barbarous husband, and this wound was followed by "convulsive startings of the arm." The same plan of treatment was pursued as in the former case, and the patient got rid of her startings. It is obvious that this case could not, with propriety, be called either trismus or tetanus. The first case was simply trismus, and, therefore, we know not why Dr. Anderson should entitle his paper—"On the Use of Tobacco in *Tetanus*." There is just barely enough of evidence here to authorize a further trial of the remedy, but nothing to engender sanguine hopes of its success.

Here we must stop for the present. For, in works of this kind, composed of various parts entirely unconnected, no inconvenience whatever is produced, even were the analytical articles as numerous as the papers in a volume of Transactions. As the design of this Journal is to give a copious and clear analysis of every work reviewed, so our readers must have patience, and not expect to have every thing at once, piping hot from the press. It is better to have a few dishes well cooked, than a medley of fragments—"the ends of all things."

[To be continued in our next.]

